# VDI pacing with temporary esophageal and transvenous pacemaker leads to treat post-cardiac surgery cardiogenic shock

**DOI:** 10.1186/s13019-022-01849-z

**Published:** 2022-05-03

**Authors:** Sameer Sharif, Adel Dyub, Craig Ainsworth

**Affiliations:** 1 Department of Medicine, Division of Emergency Medicine, Hamilton General Hospital, McMaster University, 237 Barton St East, 2nd Floor McMaster Wing, Room 252, Hamilton, ON L8L 2X2 Canada; 2 Department of Medicine, Division of Critical Care, McMaster University, Hamilton, ON Canada; 3 Department of Surgery, Division of Cardiac Surgery, McMaster University, Hamilton, ON Canada; 4 Department of Medicine, Division of Cardiology, McMaster University, Hamilton, ON Canada

**Keywords:** Pacing, Cardiogenic shock, Heart block, Post-cardiac surgery, Case report

## Abstract

**Background:**

Post-operative atrio-ventricular (AV) block after cardiac surgery is not uncommon in high-risk patients.

**Case presentation:**

Our case highlights the management of a 62-year-old female with cardiogenic shock post-cardiac surgery with concomitant complete heart block. With VVI pacing proving ineffective, it was postulated that the patient may benefit hemodynamically from AV sequential pacing, re-establishing her atrial kick. We describe a novel technique of attaching a temporary pacemaker wire to an orogastric tube to sense atrial p-waves and pace the ventricle transvenously to perform AV sequential pacing. This was done temporarily to stabilize the patient’s hemodynamic status while awaiting a permanent pacemaker implantation.

**Conclusions:**

In hemodynamically unstable post-cardiac surgery patients with complete heart block in whom VVI pacing fails to improve their clinical status, clinicians should consider VDI pacing with an orogastric atrial sensing pacemaker lead, in consultation with the cardiac surgeon and the electrophysiology team. Of note, the patient needs to have underlying organized atrial activity for this setup to work.

## Introduction

### Case

**Fig. 1:**
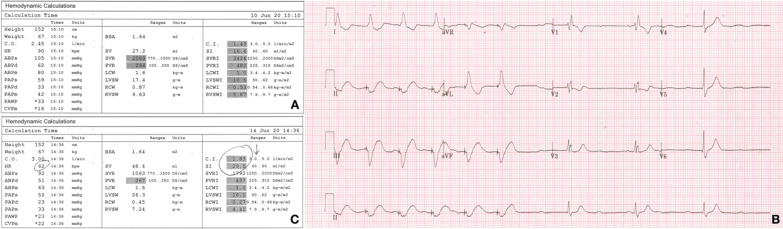
**A** Pulmonary artery catheter output of the patient in the immediate post-operative period. **B** Electrocardiogram showing underlying complete heart block with a rate of 40 beats per minute after VVI pacing paused. **C** Pulmonary artery catheter output with the patient paced VVI at rate of 62 with a transvenous pacemaker 4 days after her admission

A 63-year-old female with heart failure, coronary artery disease, mitral regurgitation, bicuspid aortic valve, and a left atrial (LA) myxoma underwent coronary artery bypass grafting surgery in addition to the myxoma resection, mechanical aortic and mitral valve replacements. She had a complicated intra-operative course whereby she decompensated after she was taken off cardiopulmonary bypass and her chest was closed. She was put back on bypass and air was removed from her right coronary artery; at this juncture, a transesophageal echocardiogram showed grossly normal left ventricular functional with reduced right ventricular function. She was admitted to the cardiac surgery intensive care unit (ICU) post-operatively and required mechanical support with an intra-aortic balloon pump (IABP). She also required norepinephrine 0.5 μg/kg/min, epinephrine 0.5 μg/kg/min, vasopressin 2 units/hour, and milrinone 0.50 μg/kg/min infusions to support her blood pressure and cardiac output (see Fig. 1A). Furthermore, she was pacer-dependent for complete heart block and was being paced via epicardial leads (VVI). Her total time on cardiopulmonary bypass was 321 min and her aortic cross-clamp time was 277 min.

On postoperative day (POD) one, cardiogenic shock persisted and the patient demonstrated significant hypoxemia. A transthoracic echocardiogram post-operatively displayed a left ventricular ejection fraction of 31% with a severely impaired right ventricle and a patent foramen ovale (PFO) with a right-to-left shunt. Prosthetic and native valve function were normal. To further aid with her hemodynamic status and manage her acidosis, she was started on inhaled nitric oxide (iNO) as well as continuous renal replacement therapy (CRRT).

With her underlying hypoxemia and the new evidence of a PFO, we removed the patient’s IABP in an attempt to improve the right atrium to left atrium pressure gradient leading to interatrial shunting. Without mechanical support, the patient’s hemodynamic status remained tenuous and she still required pacing as her underlying heart rate was in the 40 s (see Fig. 1B). We subsequently inserted a transvenous right ventricular pacemaker wire as the epicardial temporary wires inserted at the time of surgery were inconsistently capturing despite increased output settings. Despite pacing at a higher rate than the patient’s intrinsic ventricular escape rate, the patient’s cardiac indices remained poor (see Fig. 1C); of note, the patient was on similar doses of vasopressors and inotropes at this juncture.

**Fig. 2:**
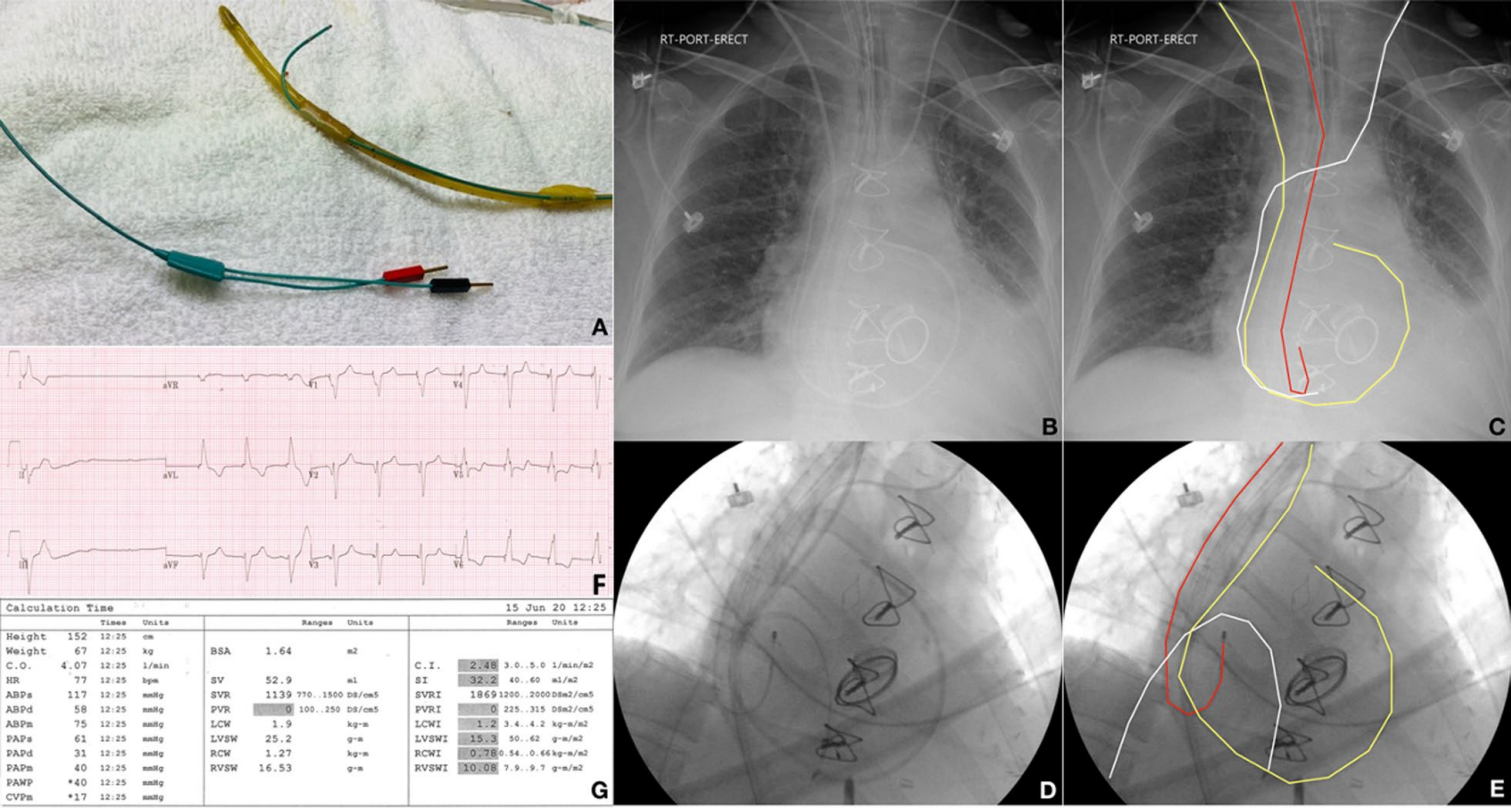
**A** Stiff wire pacemaker attached to an orogastric tube. **B** CXR of patient with a pulmonary artery catheter, endotracheal tube, mechanical mitral and aortic valve, orogastric tube, transvenous pacemaker with a left internal jugular introducer cordis, and a temporary pacemaker wire attached to an orogastric tube. **C** Same image as B with the yellow line demarcating the pulmonary artery catheter; red line demarcates the esophageal pacemaker lead; the white line demarcates the transvenous pacemaker lead. **D** Fluoroscopy image showing the pulmonary artery catheter, a transvenous pacemaker inserted through the femoral vein, and a pacemaker attached to an orogastric tube. **E** Same image as D with the yellow line demarcating the pulmonary artery catheter; the red line demarcates the esophageal pacemaker lead; the white line demarcates the transvenous pacemaker lead. **F** Electrocardiogram showing VDI pacing. **G** Pulmonary artery catheter output after the patient was being paced VDI

In an effort to further augment the patient’s cardiac output on POD 4, we attached a stiff temporary pacemaker wire to a standard orogastric (OG) tube and inserted it into the patient’s mid-esophagus. This allowed for sensing of atrial activity (p-waves) and thus permitted atrio-ventricular (AV) synchronized pacing (see Fig. 2A–F) with the goal of increasing stroke volume due to re-establishing consistent atrial contribution to ventricular filling (atrial kick). With this VDI pacing instituted, the patient’s cardiac indices improved significantly and she was able to be weaned off all of her inotropes and vasopressors (see Fig. 2G). Shortly thereafter, we replaced the transvenous pacemaker from her left internal jugular vein with a transvenous pacemaker from the left femoral vein to facilitate the insertion of a permanent pacemaker. Several days later, the patient, the patient had a cardiac resynchronization device inserted by the Electrophysiology team and she was subsequently transferred out of the ICU 8 days after her initial admission in stable condition.

## Discussion

Postoperative AV block is the most common conduction abnormality after cardiac surgery [1]. Its incidence is 1–4% depending on the procedure but can be as high as 25% after aortic valve replacement [1]. Overall, 1–5% of cardiac surgery patients require permanent pacemaker implantation [1]. Our patient had both her aortic and mitral valve replaced placing her at higher risk for developing AV block.

Post-surgery low cardiac output syndrome (LOCS) is defined as a monitored cardiac index < 2.2L/min/m^2^ with adequate or elevated cardiac filling pressures; it is often secondary to left and/or right ventricular dysfunction, but arrhythmias or valvular heart disease may also contribute [2]. Its incidence varies between 3 and 45% and is associated with the following risk factors: advanced age, prolonged bypass time, urgent surgery, and impaired left ventricular function [2]. Furthermore, myocardial revascularization, either isolated or accompanied by valve intervention, are the most frequent operations leading to LCOS [2]. Our patient had all of these risk factors. Specifically, her poor ventricular contractility, rhythm disturbance, and the air found in the right coronary artery requiring going on bypass again were the primary contributors to her LCOS.

Despite being ventricularly paced with temporary epicardial wires, our patient deteriorated requiring a transvenous pacer to maintain an adequate heart rate as her epicardial wires stopped capturing consistently. There is limited and conflicting data on the use of epicardial pacing wires after cardiac surgery as their electrical performance deteriorates over time [3]. In fact, failure to pace is observed in > 60% right and > 80% left atrial wires after 5 days [3]. The transvenous approach to pacing is often preferred as it provides superior pacing and sensing thresholds, lower lead current, and longer lead functionality [4].

This case highlights the potential importance of the atrial contribution to ventricular filling or so-called atrial kick in patients with a low cardiac output. This atrial kick contributes to about 20–30% of left ventricular end diastolic volume. As such, the loss of atrial kick can result in adverse hemodynamic consequences with a reduction in cardiac output. With the failure of conventional methods of temporary pacing in a hemodynamically unstable patient, we opted to trial a novel technique with the use of a pacemaker attached to an OG tube to sense atrial electrical activity in order to perform AV sequential pacing, immediately resulting in significant increases in the patient’s cardiac index from 1.8 to 2.5 L/min/m^2^ (see Fig. 2G). Transesophageal atrial stimulation is sometimes used in the evaluation and treatment of supraventricular arrhythmias [5]. However, its use to sense the atria and pace ventricularly through a transvenous lead has not been described in the literature.

Our case is one of the first described uses of a pacemaker lead to sense the atrial activity through the esophagus and pace the ventricle transvenously in a synchronized fashion. It is important to note that a patient needs to have underlying organized atrial activity for this setup to work. With respect to landmarking, the right atrium is located roughly 35 cm from the oral cavity down. We suggest taping the pacemaker to an OG tube and inserting it to around 35-40 cm from the oral cavity until the atrial activity is consistently sensed as identified on the external pacemaker consol. The electrophysiology team should be consulted for long-term management of these patients and the patient’s surgeon should also be informed of this maneuver being performed.

## Data Availability

Data sharing not applicable to this article as no datasets were generated or analyzed during the current study.
